# Novel roles for the radial spoke head protein 9 in neural and neurosensory cilia

**DOI:** 10.1038/srep34437

**Published:** 2016-09-30

**Authors:** Irina Sedykh, Jessica J. TeSlaa, Rose L. Tatarsky, Abigail N. Keller, Kimberly A. Toops, Aparna Lakkaraju, Molly K. Nyholm, Marc A. Wolman, Yevgenya Grinblat

**Affiliations:** 1 Department of Zoology, University of Wisconsin, Madison, WI, 53706, USA; 2 Department of Neuroscience, University of Wisconsin, Madison, WI, 53706, USA; 3 Cellular and Molecular Biology Training Program, University of Wisconsin, Madison, WI, 53706, USA; 4 Department of Ophthalmology and Visual Sciences, University of Wisconsin-Madison, Madison, WI 53706, USA; 5 McPherson Eye Research Institute, University of Wisconsin, Madison, WI, 53706, USA

## Abstract

Cilia are cell surface organelles with key roles in a range of cellular processes, including generation of fluid flow by motile cilia. The axonemes of motile cilia and immotile kinocilia contain 9 peripheral microtubule doublets, a central microtubule pair, and 9 connecting radial spokes. Aberrant radial spoke components RSPH1, 3, 4a and 9 have been linked with primary ciliary dyskinesia (PCD), a disorder characterized by ciliary dysmotility; yet, radial spoke functions remain unclear. Here we show that zebrafish Rsph9 is expressed in cells bearing motile cilia and kinocilia, and localizes to both 9 + 2 and 9 + 0 ciliary axonemes. Using CRISPR mutagenesis, we show that *rsph*9 is required for motility of presumptive 9 + 2 olfactory cilia and, unexpectedly, 9 + 0 neural cilia. *rsph*9 is also required for the structural integrity of 9 + 2 and 9 + 0 ciliary axonemes. *rsph*9 mutant larvae exhibit reduced initiation of the acoustic startle response consistent with hearing impairment, suggesting a novel role for Rsph9 in the kinocilia of the inner ear and/or lateral line neuromasts. These data identify novel roles for Rsph9 in 9 + 0 motile cilia and in sensory kinocilia, and establish a useful zebrafish PCD model.

Cilia are microtubule-based protrusions found on the surfaces of eukaryotic cells. Primary (immotile) cilia, present on most cells, receive and integrate extracellular signals^1^. Primary cilia axonemes are comprised of 9 microtubule doublets arranged around the periphery of the ciliary shaft. Motile (secondary) cilia are produced by specialized cell types and serve to generate extracellular fluid flow^2^,^3^. Studies in model organisms have produced important mechanistic insights into the early embryonic roles of motile cilia and the genetic network that underlies their formation^4^. These studies have demonstrated that, early in embryogenesis, motile cilia generate directional fluid flow in the zebrafish Kupffer’s vesicle and in the mouse node that initiates L/R asymmetry^5^,^6^. Later in development, ciliary motility is required for directional fluid flow in the pronephric ducts^7^,^8^,^9^. In the neural tube, ciliary motility is required for embryonic CSF flow and for correct morphogenesis of the brain ventricles^8^.

Two axonemal structures have been described for motile cilia: 9 + 2 axonemes, which contain 9 peripheral doublets and a central microtubule pair, and 9 + 0 axonemes, which, like primary cilia, lack the central pair. 9 + 2 cilia are associated with planar beating and are found on most multi-ciliated epithelia, including respiratory epithelia, pronephric ducts, and neural ependyma^10^. The less common 9 + 0 monocilia typically beat in a circular motion, and play crucial roles in generating left/right asymmetry and in CNS development^11^. In 9 + 2 cilia, each outer microtubule doublet is connected to the central pair via a structure called the radial spoke, a complex of 23 proteins organized into the stalk, the neck and the head of the radial spoke. The stalk forms a stable connection to the A microtubule of the outer doublets, while the spoke head connects with the central pair^12^,^13^,^14^,^15^.

Defects in 9 + 2 motile cilia formation result in primary ciliary dyskinesia (PCD), an autosomal recessive disorder that affects an estimated 1 in 15,000 births. PCD is clinically heterogeneous, presenting with chronic respiratory infections, hearing impairment, infertility, *situs inversus* and hydrocephalus^16^. To date, mutations in at least 31 genes have been identified as causative of PCD, accounting for about 70% of disease incidence^17^,^18^,^19^,^20^,^21^,^22^,^23^,^24^,^25^,^26^. These genes encode cytoplasmic proteins important for ciliary assembly, as well as components of the dynein arms, the central microtubule pair, and radial spokes^27^. Thus far, four proteins of the spoke head have been associated with PCD in humans: RSPH1, RSPH3, RSPH4A and RSPH9 23,28,29,30,31,32. Patients with spoke head mutations typically have ciliary transposition defects characterized by loss of the central pair and displacement of an outer microtubule doublet into the center of the axoneme^23^,^28^,^31^. Airway cilia from these patients show partially penetrant deficits in beat frequency and waveforms, which range from normal (planar) to aberrant (rotational), suggesting that radial spokes are involved in setting the parameters of ciliary motility^23^,^30^.

Due to their association with the central microtubule pair, radial spoke head protein function has been presumed to be restricted to 9 + 2 motile cilia. Support for this hypothesis was recently provided by Shinohara *et. al*.^33^, who showed that mouse embryos with rsph4a mutations develop with aberrant airway cilia, but normal 9 + 0 motile cilia of the node. In this report, we use genetic and imaging tools to examine the role of the radial spoke head component Rsph9 in the 9 + 2 olfactory motile cilia, 9 + 0 neural motile cilia and in 9 + 2 immotile kinocilia. We show that Rsph9 is required for the correct motility in both types of motile cilia, and present indirect evidence suggesting a functional role for Rsph9 in kinocilia-bearing hair cells.

## Results

### Radial spoke head component Rsph9 is expressed in motile ciliogenic cells in zebrafish embryos

**Figure 1:**
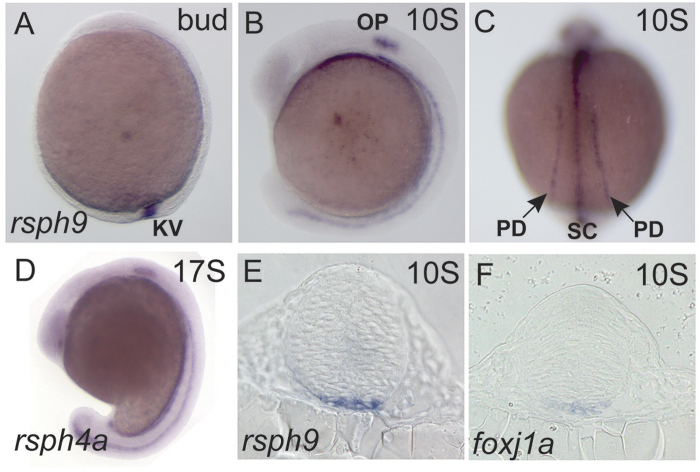
Rsph9 and Rsph4a are expressed in the motile ciliogenic embryonic domains. Wild-type embryos were stained by ISH to identify cells that express *rsph*9 (**A**–**C**,**E**), *rsph*4a (**D**) and *foxj*1a (**F**). (**A,B,D**) are whole mount embryos in lateral view, anterior to the left. (**C**) Is a dorsal view. (**E**,**F**) are transverse sections at the level of midbrain. KV: Kupffer’s vesicle; OP: otic placode; PD: pronephric duct; SC: ventral spinal cord.

In zebrafish, motile cilia are produced by Kupffer’s vesicle (KV, the zebrafish equivalent of the mammalian node), the pronephric ducts, the nasal and otic placodes, and ventral spinal cord^34^,^35^,^36^. As in other vertebrates, most motile cilia have the canonical 9 + 2 axonemes with the exception of those in the spinal cord, where 9 + 0 cilia predominate^8^. Since expression of radial spoke components has not been described in zebrafish, we isolated cDNA clones that encode Rsph9 and Rsph4a (see Materials and Methods) and determined their expression patterns using whole mount *in situ* hybridization (WISH). *rsph*9 was expressed in the ciliated structures, namely, KV (Fig. 1A), pronephric ducts, otic placodes, and ventral spinal cord (Fig. 1B,C). *rsph*4a was expressed in a similar pattern (Fig. 1D). *rsph*9 was also expressed in the ventral midline of the midbrain primordium (Fig. 1C,E), as was *foxj1a* (Fig. 1F), a key transcriptional regulator of motile ciliogenesis^37^,^38^,^39^.

**Figure 2:**
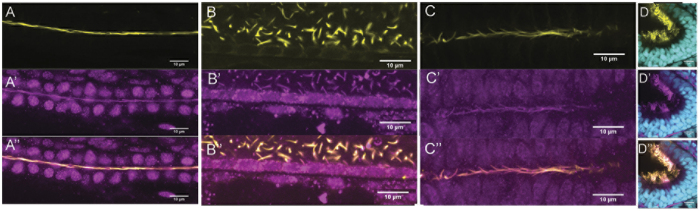
Rsph9 protein is enriched in cilia. Wildtype embryos were stained with an anti-Rsph9 antibody (magenta) and acetylated α-tubulin (yellow). (**A**) Pronephric duct. (**B**) Ventral spinal cord. (**C**) Ventral midbrain. (**D**) Olfactory pit, nuclei are visualized with DAPI (cyan). All images are shown with anterior to the left. (**A,B**) Are lateral mounts (dorsal at the top).

We next asked if zebrafish Rsph9 protein localizes to the ciliary axonemes, using an antibody that recognizes a conserved epitope in *Chlamydomonas reinhardtii* and human RSPH9 proteins (see Methods). Co-immunostaining with acetylated tubulin, which marks ciliary axonemes, revealed Rsph9 reactivity along the length of most or all motile ciliary axonemes, including cilia of the pronephric ducts, the spinal canal, the ventral midbrain and olfactory pits (Fig. 2A–D, Supp. Fig. 1).

### Olfactory cilia require Rsph9 for normal motility

**Figure 3:**
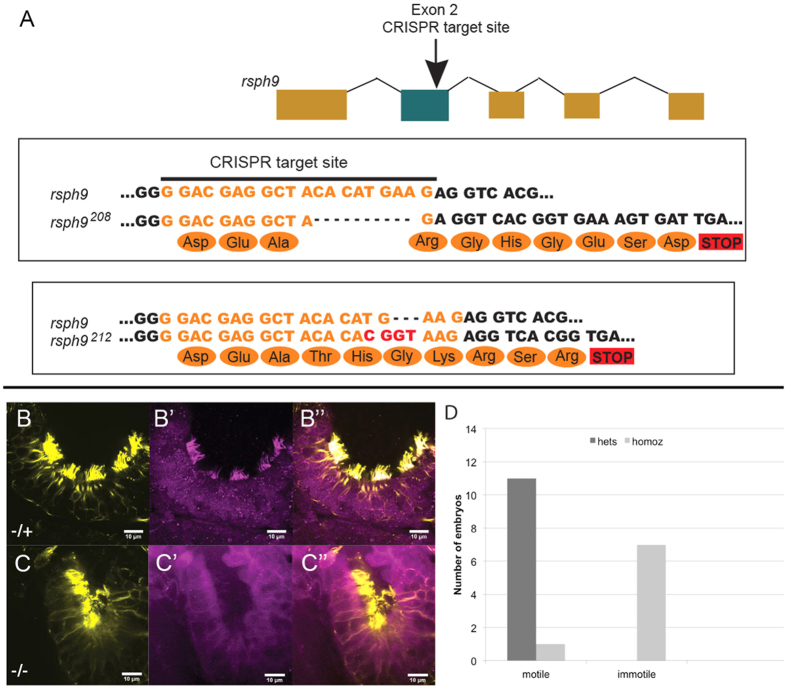
Olfactory cilia require Rsph9 function for motility. (**A**) Two independent mutant alleles at the *rsph*9 locus were obtained using CRISPR/Cas9 mutagenesis. (**B**,**C**): *rsph*9^208^/+ (**B**) and *rsph*9^208^/*rsph*9^208^ (**C**) siblings were fixed at 5 dpf and stained for acetylated α-tubulin (yellow) and Rsph9 (magenta). (**D**) Chart summarizing olfactory ciliary motility shows reduced motility in homozygous mutants.

To ask if Rsph9 function is required for ciliary motility in zebrafish, we used CRISPR-Cas9 targeted mutagenesis to generate mutations in exon 2 of the Rsph9 locus (see Materials and Methods for details). Two mutant alleles were isolated that contain, respectively, an 8 bp deletion (*rsph*9^208^) or an indel with a net gain of 2 bp (*rsph*9^212^) at the CRISPR target site. Both mutations are predicted to result in a frame shift and a truncated protein (Fig. 3A; Supp. Fig. 2). Western blot analysis confirmed the presence of a 30 kD band recognized by Rsph9 antibody in +/+ and *rsph*9^208^/+ embryos, and the absence of this band in *rsph*9^208^/ *rsph*9^208^ siblings (Supp. Fig. 2). Homozygous mutant embryos also lack Rsph9 immunoreactivity in the olfactory cilia, further confirming absence of full-length Rsph9 (Fig. 3C,D).

The olfactory epithelium contains multiciliated cells with 9 + 2 motile cilia^40^,^41^ and is easily accessible for live imaging. Rsph9 homozygous zebrafish formed normal olfactory structures by day 4 dpf (Fig. 3C). Motility of the olfactory cilia was visualized by high-speed (240 fps) live imaging in a group of *rsph*9^208^ homozygous and heterozygous siblings, genotyped post-hoc (Supp. Movies OC1-4, Supp. Fig. 3). This analysis, summarized in Fig. 3D and Supp. Table 1, revealed robust, coordinated ciliary motility in all tested *rsph9*^*208*^/+ larvae (11 total) and greatly diminished motility in 8 out of 9 *rsph9*^*208*^/ *rsph9*^*208*^ larvae. These results are consistent with a functional requirement for zebrafish Rsph9 in 9 + 2 cilia with radial spokes.

### Rsph9 is required for motility of 9 + 0 cilia in the ventral spinal cord

**Figure 4:**
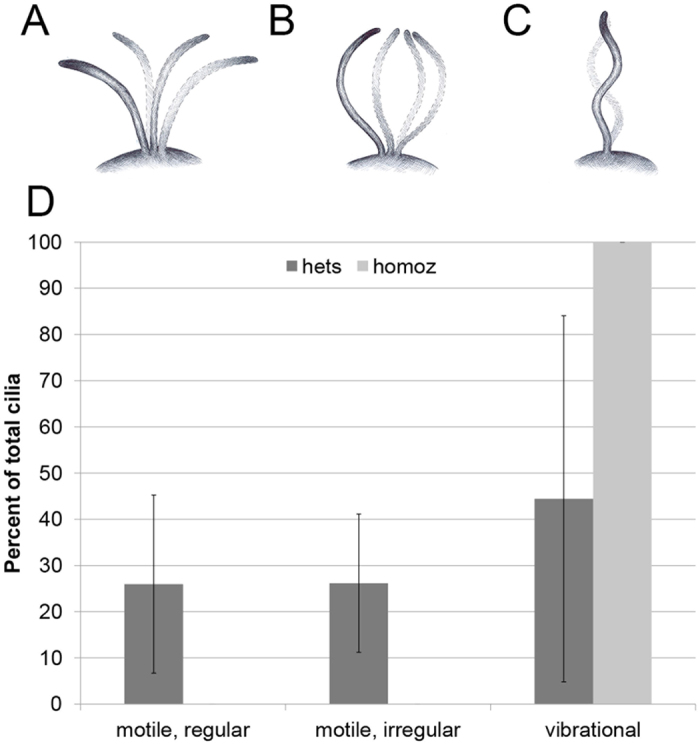
Motile spinal cord cilia require Rsph9 function. (**A**–**C**) schematics illustrating the three observed motility patterns: regular (**A**) irregular (**B**) and vibrational (**C**). (**D**) Neural ciliary motility is impaired in homozygotes (n = 15/19 cilia, 2 embryos) vs. heterozygous siblings (n = 54/55 cilia, 3 embryos). Bars = standard deviation.

We next asked if Rsph9 is also required for the motility of ventral spinal cord cilia, previously reported to have 9 + 0 axonemes^8^. *Rsph*9^208^ homozygotes with fluorescently labeled spinal cord cilia were produced by crossing *Tg(β-actin:Arl13b-GFP)*^42^ into the *rsph*9^208^ mutant background. Embryos derived from a cross between *rsph*9^208^/*rsph*9^208^ males and *rsph*9^208^/+; *Tg(β-actin:Arl13b-GFP)*/+ females were selected for GFP fluorescence. Five embryos were imaged live using high-speed confocal microscopy, with multiple individual cilia scored for motility in each (see Methods, Supp. Movies SC1-5, and Supp. Table 2). Heterozygous siblings contained a mixed population of cilia that moved vigorously in a regular waveform (Fig. 4A), in an irregular pattern (Fig. 4B), or remained largely immotile, with a slight motion that we described as vibrational (Fig. 4C). In contrast, only the vibrational, largely non-motile cilia were observed in the spinal cords of homozygous mutants (Fig. 4D). Taken together with the previously reported 9 + 0 axoneme structure^8^ and ciliary localization of Rsph9 in the ventral spinal cord (Fig. 2), these findings indicate a novel role for Rsph9 in 9 + 0 motile cilia.

### Rsph9 is required for structural integrity of 9 + 0 and 9 + 2 motile cilia

In PCD patients with RSPH mutations, respiratory epithelial cilia show characteristic ultrastructural defects, namely, loss of the central microtubules and transposition of one of the outer doublets to the center of the axoneme^23^,^28^,^29^,^30^,^31^,^32^. To determine if Rsph9 is similarly required for ciliary structure in zebrafish, we examined *rsph*9 mutant embryos using transmission electron microscopy (TEM).

**Figure 5:**
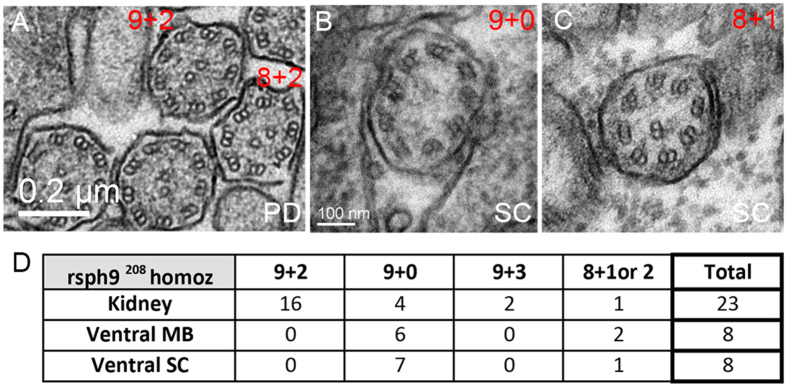
Ultrastructural defects in *rsph*9 mutant cilia. (**A**–**C**): Representative TEM images of cilia from *rsph9*^*208*^*/ rsph9*^*208*^ embryos at 1 dpf. Both the normal 9 + 2 and aberrant 8 + 2 axonemes are present in the pronephric ducts (**A**). Normal 9 + 0 (**B**) and aberrant 8 + 1 (**C**) cilia are found in the ventral spinal cord. (**D**) Summary of ciliary structures observed by TEM in *rsph9*^*208*^*/ rsph9*^*208*^ embryos surveyed at kidney level (5 embryos, 2 experiments), midbrain level (3 embryos, 1 experiment), and spinal cord level (4 embryos, 2 experiments). PD: pronephric duct; SC: ventral spinal cord.

We first asked if 9 + 2 cilia in the pronephric ducts form aberrantly in *rsph*9 homozygous zebrafish. These embryos develop without overt phenotypic deficits and are adult viable. Nonetheless, pronephric ducts of *rsph9* homozygotes formed cilia with a variety of aberrant axonemes (7 aberrant out of 23 total, Fig. 5A). Next, we assayed ciliary structure in the ventral neural tube of *rsph*9 mutant embryos at 1 dpf. In the spinal cord, 7 out of 8 cilia had the expected 9 + 0 axonemes (Fig. 5B), while one had an aberrant 8 + 1 structure (Fig. 5C). In the anterior neural tube (ventral midbrain), *rsph9* homozygotes also contained a mixture of normal 9 + 0 and aberrant 8 + 1 axonemes (2 aberrant out of 8 total; Fig. 5D). To corroborate specificity of this aspect of the mutant phenotype, we examined ciliary structures in embryos depleted of Rsph9 by antisense morpholino injection (Supp. Fig. 4). Neural tube cilia in Rsph9 morphants presented with a mixture of normal 9 + 0 axonemes and aberrant 8 + 1 configurations (Supp. Fig. 5). Likewise, ~ half of the morphant pronephric duct axonemes were aberrant (Supp. Fig. 5).

**Figure 6:**
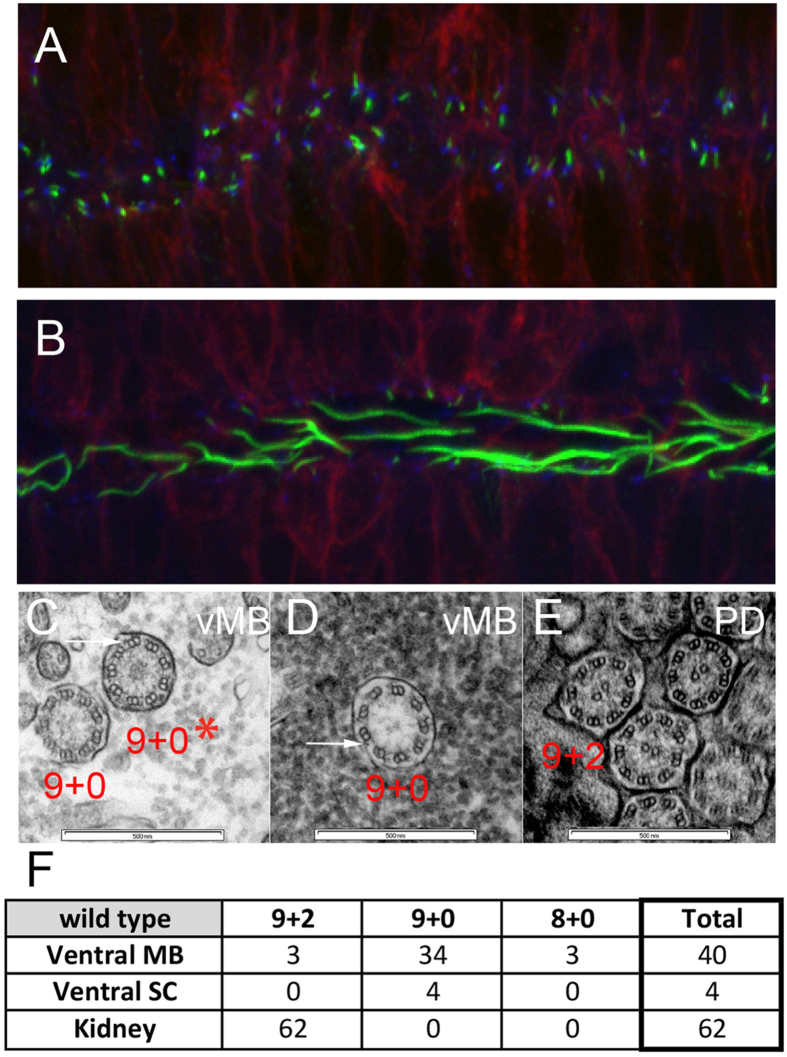
Ventral midbrain and ventral spinal cord produce long cilia with 9 + 0 axonemes. (**A**,**B**) *Tg(β-actin:mGFP)* embryos at 24 hpf were stained with antibodies against acetylated α-tubulin (green) and γ-tubulin (blue) to label ciliary axonemes and basal bodies, respectively. (**A**) Short cilia at the dorsal midbrain midline. (**B**) Long cilia at the ventral midbrain midline. (**C**–**E)** Wildtype embryos at 1 dpf were processed for transmission electron microscopy and analyzed in transverse sections at the levels of ventral midbrain (**C**,**D**) and pronephric duct (**E**). Arrows point to outer dynein arms. (**F**) Summary of ciliary structures in the ventral midbrain, spinal canal, and pronephric ducts of wiltype embryos.

Since to date motile neural cilia have been characterized only in the posterior neural tube, i.e. spinal cord, we wished to confirm the presence of 9 + 0 cilia in the anterior neural regions during normal development. Cilia in the dorsal midline of the brain primordium (midbrain level) were uniformly short, as expected for non-motile primary cilia (Fig. 6A; Supp. Fig. 6). In contrast, cilia produced at the ventral midline, which expresses *rsph*9 and *foxj*1a, were substantially longer and continued to elongate in the course of development (Fig. 6B; Supp. Fig. 7). A range of axonemal structures was revealed by TEM analysis of ventral midbrain cilia in 1 dpf wildtype embryos, with many lacking the central microtubule pair (34 of 40; Fig. 6C,D). Outer dynein arms were visible in 9 + 0 cilia, consistent with putative motility (arrows in Fig. 6C,D). Pronephric ducts contained only the typical 9 + 2 axonemes (Fig. 6E,F), indicating that we were able to consistently visualize 9 + 2 axonemal structures. Collectively, these data strongly suggest that Rsph9 function contributes to the structural integrity of ciliary axonemes in both 9 + 2 and 9 + 0 cilia, and that structural ciliary defects in *rsph*9 mutants have functional consequences for 9 + 0 ciliary motility.

### Behavioral assays identify a role for Rsph9 in acoustic sensory reception

**Figure 7:**
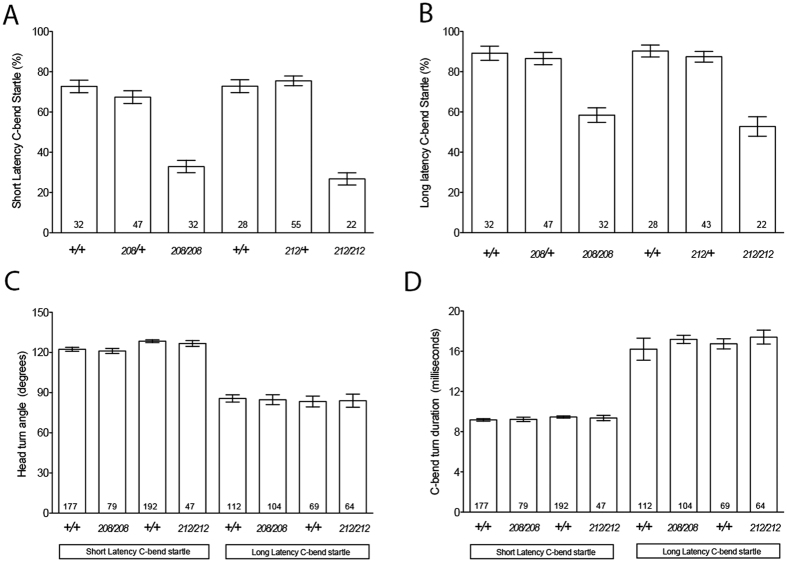
Initiation of acoustic startle response is impaired in Rsph9 mutants. (**A**) Initiation of the short-latency curve (SLC) maneuver in response to high intensity stimuli is reduced in *rsph*9^208^/*rsph*9^208^ and in *rsph*9^212^/*rsph*9 ^212^ larvae compared to homozygous wild type and heterozygous siblings. (**B**) Initiation of the long latency C-bend (LLC) escape maneuver is reduced *rsph*9^208^/*rsph*9^208^ and in *rsph*9^212^/*rsph*9 ^212^ larvae compared to siblings. (**C**) Head turn angle during ASR to high-stimulus is not dependent on the *rsph*9 genotype. (**D**) Turn duration (in milliseconds) during ASR to high-stimulus is not dependent on Rsph9 genotype. Error = SEM. N larvae at bottom of bar. *p < 0.001 ANOVA vs wildtype siblings.

*rsph*9 is expressed in the otic primordium, where it localizes to kinocilia (Supp. Fig. 8). Hair cells of *rsph*9 mutant larvae form kinocilia (Supp. Fig. 9) and contain functional mechanotransduction channels, indicated by the ability of hair cells to take up the vital dye FM1-43 (Supp. Fig. 9). To test function of hair cells that lack Rsph9, we measured the ability of *rsph*9 mutant larvae to perform a startle response to acoustic stimulation using a behavioral platform that provides rigorous quantitative assessment of initiation and execution of this highly stereotyped behavior^43^,^44^. Individual larvae derived from an *rsph*9^212^/+ incross were exposed to a series of 20 non-habituating acoustic stimuli of low or high intensity and then genotyped post-hoc. *rsph*9^212^/*rsph*9^212^ larvae showed reduced initiation of both the short latency and long latency startle responses to high-intensity stimuli (Fig. 7A,B and Supp. Table 3) as well as to low intensity stimuli (data not shown). A similar deficit was observed in *rsph*9^208^/*rsph*9^208^ larvae (Fig. 7A,B and Supp. Table 3). Importantly, homozygous mutant larvae that did initiate a startle response performed it normally (Fig. 7C,D), indicating that spinal motor components of the startle circuit are functional in *rsph*9 homozygotes. Moreover, reduced excitability of hindbrain interneurons, such as the Mauthner neuron, result in deficits in the short, but not the long-latency startle responses^43^. Since the *rsph*9 homozygous mutants show reduced initiation of both the short and long-latency startle response, the behavioral impairment is likely due to a sensory impairment, rather than a defect in the startle circuitry. Collectively, these results argue that the behavioral deficit in *rsph*9 mutant larvae lies upstream of the motor circuits which coordinate startle response performance, and are likely attributable to acoustic sensory impairment.

## Discussion

**Figure 8:**
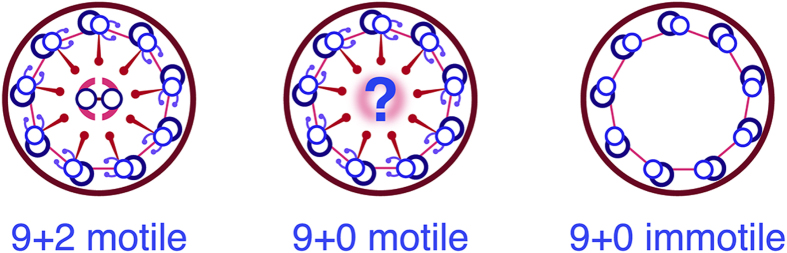
Schematic comparison between motile 9 + 2, motile 9 + 0 and primary (immotile) 9 + 0 ciliary structures. The model diagrammed here suggests a close structural relationship between motile 9 + 0 cilia and motile 9 + 2 cilia. Specifically, 9 + 0 motile cilia are proposed to contain radial spokes that interact with a putative complex of proteins in the center. This complex may be related to the protein sheath that surrounds the central microtubule pair in 9 + 2 cilia.

*Rsph*9, a structural component of radial spokes found in motile 9 + 2 cilia, is mutated in a subset of PCD patients. Here we take advantage of the accessibility of embryonic zebrafish to examine *rsph*9 function in 9 + 0 vs 9 + 2 motile cilia *in vivo*. Our data demonstrate that zebrafish Rsph9 protein localizes to both types of motile cilia, and that depletion or absence of Rsph9 causes structural defects in neural 9 + 0 and pronephric 9 + 2 cilia. Remarkably, these defects closely resemble those reported in 9 + 2 cilia on respiratory epithelia from PCD patients with RSPH9 mutations. While radial spokes are known to interact with the central microtubule pair in 9 + 2 cilia, their involvement in 9 + 0 motile cilia was unexpected. A plausible explanation consistent with our data is that, even in the absence of central microtubules, motile cilia contain a modified central sheath complex that interacts with radial spokes. As in 9 + 2 cilia, we hypothesize that this interaction is necessary for keeping the outer microtubules properly aligned, and for the correct motility of 9 + 0 cilia. Testing this hypothesis, diagrammed in Fig. 8, will require identification of additional central complex components and determining their functions in motile ciliary subtypes in accessible model organisms.

It is possible that the absence of the central pair in neural cilia is an artifact of tissue processing that fails to preserve this structure. In this case, the electron-dense center visible in many 9 + 0 axonemes may be a remnant of the central pair. This concern is partially alleviated by our ability to see perfectly preserved central microtubule pairs in pronephric duct cilia, as well as centrally located microtubule singlets in aberrant neural cilia. Nonetheless, the definitive test will require additional ultrastructural examination of 9 + 0 cilia with more sensitive methods, e.g. the recently developed cryo-electron tomography^45^.

Presumptive motile cilia are present on the apical surface of the neuroepithelium even before the lumen opens and begins to fill with cerebrospinal fluid (CSF) at ~24 hpf, and well before the CSF-producing choroid plexus forms at 48 hpf^46^. The physiological significance of motile cilia at this early stage is unclear. It is widely assumed that motile cilia move fluid through the neural lumen similar to the way they mediate directional fluid flow in the pronephric duct. However, 9 + 0 cilia in the embryonic neural tube do not appear to beat coordinately and are unlikely to generate long-range directional flow. Instead, they may be important for local re-distribution of CSF components, i.e. growth factors. Later in development, motile ciliary dysfunction is linked to hydrocephalus, a debilitating symptom of PCD that is caused by excess cerebrospinal fluid (CSF) in the brain lumen. Excess CSF can result from overproduction of CSF, impaired reabsorption of CSF, or blockage of one of the narrow foramina connecting the ventricles (frequently the cerebral aqueduct). The underlying mechanisms that lead to CSF accumulation in PCD patients are poorly understood. *Dnah*5 mutant mice have dysmotile cilia and reduced CSF flow velocity, which is thought to cause occlusion of the cerebral aqueduct and fluid buildup^47^,^48^. In contrast, ciliary dysmotility in zebrafish *rsph*9 homozygotes and PCD patients with RSPH mutations is not associated with hydrocephaly and does not preclude normal development, likely due to the fact that Rsph-deficient cilia retain some motility.

The bulk of ciliary structure-function studies have been performed in the alga *Chlamydomonas reinhardtii*, whose cilia are exclusively 9 + 2. Based on these studies, radial spokes are thought to transduce signals from the central pair to the outer doublets, where asymmetrical dynein activity is responsible for driving ciliary beating^49^,^50^. Radial spokes are also thought to be involved in controlling the waveform, which in 9 + 2 cilia is usually planar. There are exceptions to this rule, e.g. in the zebrafish Kupffer’s vesicle (KV), where both 9 + 2 and 9 + 0 cilia that beat with a rotational waveform have been described^8^,^51^,^52^,^53^. Proper motility of KV cilia is required for correct L/R asymmetry during zebrafish embryogenesis, yet *rsph*9^208^ and *rsph*9^212^ homozygotes do not exhibit L/R deficits (data not shown). This observation suggests that Rsph9 function is not absolutely required for KV cilia motility, but does not rule out a role for Rsph9 in modulating motility. How radial spokes contribute to controlling motility in KV cilia, particularly to the choice of waveform, is an important outstanding question, and the zebrafish *rsph*9 mutants will be instrumental in arriving at the answer.

Respiratory dysfunction, which includes neonatal respiratory distress and severe sinusitis, is the most prominent clinical presentation in patients with Rsph-linked PCD. Nasal biopsy scrapings are commonly used as an accessible proxy for respiratory passage lining to assess ciliary motility in PCD patients^54^. While zebrafish lack airway epithelia, their non-sensory olfactory epithelium is also multi-ciliated and bears 9 + 2 motile cilia that beat in a coordinated fashion, creating directional flow of the mucus that leads to odorant exchange between the olfactory pit and the environment^40^,^41^,^55^. Zebrafish olfactory epithelium is superficially located, accessible to high-resolution live imaging in intact animals, and eminently suitable for use in high-throughput genetic and small molecule screens.

Zebrafish process high-frequency auditory input that activates acoustic startle response primarily through the sacculae of the inner ear; importantly, this machinery is already in place by 5 dpf, when our behavioral assays were conducted^56^. Acoustic response deficits in larvae that lack full-length Rsph9 are consistent with a requirement for radial spokes in the inner ear, specifically in the immotile 9 + 2 kinocilia produced by the hair cells. PCD patients with radial spoke defects suffer from hearing deficits thought to be caused by impaired mucosal clearance in the middle ear^57^; consequently, the potential role for radial spokes in kinocilia of the inner ear has not been addressed.

Kinocilia play essential, conserved roles in organizing stereociliary bundle formation^58^,^59^,^60^. Kinocilia also have direct mechanosensory functions in some contexts, e.g. the nascent hair cells in the zebrafish lateral line. The zebrafish inner ear contains an additional ciliary subtype with a 9 + 2 axoneme: motile cilia that form adjacent to kinocilia at the poles of the otic placode. These cilia play an auxiliary role in otolith biogenesis^61^,^62^,^63^. *rsph*9 mutants develop with normal otolith numbers, position and morphology (data not shown), implying that Rsph9 is not required for motility of otic cilia. Together with the apparently normal otolith morphology, acoustic deficits in *rsph*9 mutant larvae point to a potential novel function for Rsph9 in hair cell kinocilia. The robust startle response defect in *rsph*9 mutant larvae, combined with our ability to perform high throughput, behavior-based small molecule screens in zebrafish^44^, constitute a powerful tool for discovery of drugs that attenuate sensory impairment in an *in vivo* PCD model.

There is still much to understand about how radial spokes regulate ciliary motility during vertebrate embryogenesis, particularly in the developing neuroepithelium, where 9 + 0 motile cilia predominate. The *rsph*9 mutant zebrafish described here represent a novel tool for dissecting the mechanisms of radial spoke head function in a range of ciliary subtypes. This mutant line is also ideally suited for testing therapeutic approaches to alleviate mechanosensory and motility deficits that contribute to the severity of PCD, e.g. small molecule screening or mRNA therapeutics, an active area of research in respiratory disease treatment^64^.

## Materials and Methods

### Zebrafish strains and embryo manipulation

Adult zebrafish were maintained according to established methods^65^. All experimental protocols using zebrafish were approved by the University of Wisconsin Animal Care and Use Committee, and carried out in accordance with the institutional animal care protocols. Embryos were obtained from natural matings and staged according to ref. 66. Transgenic *Tg(β-actin:Arl13b-GFP)* zebrafish, kindly provided by Brian Ciruna, were used to generate embryos with fluorescently labeled cilia^42^. Gene-specific antisense oligonucleotide morpholinos were purchased from GeneTools (Philomath, OR) and included Rsph9 translation-blocking morpholino (rsph9MO, Castleman *et al*., 2009), p53 MO and standard control MO (conMO). 1–2 nl of Rsph9 or control MOs (2–4 ng/nl) were injected singly or in combination with p53 MO (4–6 ng/nl) in HEPES/KCl buffer^67^ into 1–2 cell stage embryos. For testing hair cell mechanotransduction, larvae were incubated in E3 with 3 uM FM1-43 for 45 seconds, rinsed twice for 5 minutes and imaged using a Leica stereoscope.

### Immunohistochemistry, histology and *in situ* hybridization (ISH)

Embryos for immunohistochemistry were fixed in 4% paraformaldehyde in PBS, subjected to antigen retrieval according to ref. 68 except that TritonX-100/goat serum were used in place of Tween/sheep serum. For Western analysis, individual embryos were genotyped from larval tailclips and protein lysates were extracted at 5 dpf according to ref. 69. The following antibodies were used: rabbit anti-Rsph9 (1:200 for IHC, 1:250 or 1:430 for Western blot, Sigma HPA031703), mouse anti-b-actin (1:5,000, Sigma A1978), mouse anti-acetylated tubulin (1:400, Sigma T6793), anti-gamma tubulin (1:1000, Sigma), mouse anti-GFP (1:500, Chemicon), rabbit anti-GFP (Life Technologies), and anti-tRFP (1:500, Evrogen). For IHC, primary antibodies were detected fluorescently with Alexa-labeled goat anti-mouse or goat anti-rabbit secondary antibodies (1:1000, Molecular Probes). For Western analysis, goat anti-rabbit secondary HRP conjugated antibodies were used (1:2,500, Promega W401B) and goat anti-mouse HRP (1:10,000, ThermoScientific 31430). Nuclei were counterstained with DAPI (Invitrogen). Embryos were mounted in VectaShield and imaged on an Olympus IX81 inverted confocal microscope with the Fluoview 1000 confocal package, using a 60x water immersion objective (NA 1.10) or 60x oil immersion objective (NA 1.35).

WISH was carried out as previously described^70^. Stained embryos were embedded in Eponate 12 (Ted Pella) and 5–7 μm sections were cut with a steel blade on an American Optical Company microtome. Antisense digoxigenin-labeled RNA probes were transcribed using the MAXIscript kit (Ambion).

*rsph*9 and *rsph*4a cDNAs were amplified from total cDNA of 24 hpf embryos by PCR with the following primers: rsph4a (Sense: 5′-ATGGAGATTACAGGTGAAGCG-3′, Antisense: 5′-TTTGGCACTGATGCAGATGG-3′); rsph9 (Sense: 5′-ATGGACTCTGATTCTCTG-3′, Antisense: 5′-GATTGTGTCGCTGAAGTC-3′), and TA-cloned into pGEMT-Easy (Promega). Probes for WISH were generated by transcription from these plasmids, and from *foxj*1a^38^.

### Transmission electron microscopy

Yolk cells of segmentation-stage embryos were injected with 6–8 nl of 40 mM AMP-PNP and yolk was expelled several minutes later using tweezers and a fine-gauge needle (adapted from^71^. Embryos were then transferred to a glass petri dish with glutaraldehyde fix in 200 mM phosphate buffer, dissected in two just behind the otic placode and processed for TEM according to Jaffe *et al*., 2010.

### CRISPR mutagenesis and high-resolution melt analysis (HRMA)

The target site in exon 2 of rsph9 was selected using the ZiFiT Targeter website (http://zifit.partners.org/) with the criteria: 5′GG(N)18NGG3′. Guide RNA expression vectors were constructed and transcribed as described in ref. 72 with the target sequence 5′GGACGAGGCTACACATGAAG5′. 2 nL of a mix containing 25 ng/μL sgRNA and 450 ng/μL Cas9 mRNA was injected into 1-cell embryos. Genomic DNA was extracted from single embryos or adult fish tail clips according to ref. 73. qPCR amplification was performed on a StepOnePlus system (Life Technologies). Each 20 μL reaction contained a final concentration of 0.2 μM primers, 1X MeltDoctor HRM Master Mix (Life Technologies), and 2 μL gDNA. Primers were designed using the PrimerExpress software (Life Technologies) to amplify a 105 base region spanning the 3′ end of exon 2. Primer pairs were tested for efficiency as in ref. 74. Each sample was loaded with three technical replicates on a single plate. rsph9R ex2 CRISPR primer sequences were 5′ACCCGTCCCATGAGTACGA3′ in rsph9 exon 2 and 5′GCCCATCTGTGTGGTGTAAGG3′ in rsph9 intron 2–3 (IDT). Melt curve analysis was performed using the MeltDoctor High Resolution Melt v3.0 Software. Melt profiles were automatically sorted into variant groups by the software based on melting temperature and melt curve shape. Samples were manually determined to be wildtype, heterozygous, or homozygous mutant at the *rsph*9 locus.

### Sequencing and PCR genotyping of rsph9 mutant alleles

PCR fragments identified as mutant by HRMA were subcloned via TA cloning into pGEMT-Easy (Promega) and sequenced to characterize the mutant alleles. Subsequently, to efficiently genotype individual embryos and adult fish, PCR (see above for primer sequence) followed by Metaphor gel electrophoresis were used to identify *rsph*9^208^ allele. A digest with MboII (NEB) was added before electrophoresis to identify *rsph*9^212^ allele (see Supp. Fig. 2)

### Imaging ciliary motility in the spinal cord

Live imaging of spinal cord cilia was performed on the Revolution XD spinning-disk microscopy system (Andor, Belfast, UK) equipped with the Yokogawa CSU-X1 confocal spinning disk head (Yokogawa Electric Company, Tokyo, JP); Nikon Eclipse Ti inverted microscope (Nikon, Melville, NY); iXon x3 897 EM-CCD camera (Andor); Andor laser combiner with four solid-state lasers at 405, 488, 561, and 640 nm and corresponding band-pass filter sets (Sutter, Novato, CA); and ASI motorized stage with piezo-Z for rapid Z-stack acquisition (Applied Scientific Instrumentation, Eugene, OR). Images were acquired using either the 60x/1.4 NA Plan Apo VC or 100 ×/1.49 NA Apo TIRF objectives (Nikon, Melville, NY) for 50–150 frames at ~20 frames per second. During image acquisition, the same laser power, exposure and electron-multiplying gain settings were kept for each animal. Andor IQ3 software (Andor) was used for image acquisition and Imaris ×64 v. 7.1 (Bitplane, Zurich, CH) for conversion to movies. Imaris software (Bitplane) was used for background subtraction (using the background-subtraction algorithm and identical automatic threshold for all images), smoothing (Gaussian algorithm with identical threshold settings for all images), and conversion of the images to movies. 20 fps is just above the reported beat frequency of spinal cord cilia, ~12 beats per second^8^. It is insufficient for rigorous quantification^34^ but allows qualitative assessment of ciliary motility. Individual cilia were scored qualitatively by two of the authors independently and in a genotype-blind manner, i.e. before embryonic genotypes were determined.

### Olfactory cilia imaging

For direct visualization of ciliary motility, 4 dpf embryos were mounted in 3% methylcellulose and imaged on the upright Nikon Eclipse E600 microscope with a 60x NA:0.95 objective, using a Apple iPhone 6 in slo-mo mode (240fps), mounted with a iDu professional iPhone 6 microscope adapter with built-in 30 mm 10x WF lens. Ciliary movement in the nasal pits was recorded for ~10s without digital zoom, then for ~10s with digital zoom. Movies (Supp. Movies OC1-19) were played back at 24 fps (0.1x of the original speed) and analyzed qualitatively for normal versus reduced ciliary motility, blind to genotype, i.e. prior to identifying homozygous mutants by PCR.

### Behavioral testing

All behavioral testing was conducted in a custom-built, computerized programmable system consisting of (1) a grid, where 16 individually housed larvae are tested simultaneously; (2) a vibrational mini-shaker, which delivers vibrational/acoustic stimuli of defined strength (1000 Hz) to the grid; (3) a high-speed camera that records the behavioral responses at 1,000 frames/second, and (4) a computer with custom software, which allows customized testing regimes to be programmed. Acoustic stimuli produced by the system range from low, subthreshold ones that do not elicit a response to robust, above-threshold levels that evoke an explosive startle response. Optical stimuli are delivered by briefly turning off the light (“dark flash”). After recording behavioral responses, movement tracks of each individual larva are analyzed frame by frame, automatically reconstructed over time, and the kinematic parameters of the response are calculated to describe the behavior^43^.

## Additional Information

**How to cite this article**: Sedykh, I. *et al*. Novel roles for the radial spoke head protein 9 in neural and neurosensory cilia. *Sci. Rep.* **6**, 34437; doi: 10.1038/srep34437 (2016).

## Supplementary Material

Supplementary Information

Supplementary Information

Supplementary Information

Supplementary Information

Supplementary Information

Supplementary Information

Supplementary Information

Supplementary Information

Supplementary Information

Supplementary Information
