# Supplementary material for: Novel roles for the radial spoke head protein 9 in neural and neurosensory cilia
Source: Sci Rep. 2016 Sep 30;6:34437. doi: 10.1038/srep34437 (PMC5043386; doi:10.1038/srep34437)
Supplement: Supplementary Information [file srep34437-s10.pdf]

Supplementary Information

Novel roles for the radial spoke head protein 9 in neural and neurosensory cilia.

Irina Sedykh[1,2], Jessica J. TeSlaa[1,2,3,6], Rose L. Tatarsky[1,2], Abigail N. Keller[1,2,7], Kimberly A. Toops[4,5], Aparna Lakkaraju[4,5] , Molly K. Nyholm[1,2,8], Marc A. Wolman[1], Yevgenya Grinblat[1,2,5] *

[1] Department of Zoology, University of Wisconsin, Madison, WI, 53706, USA

[2] Department of Neuroscience, University of Wisconsin, Madison, WI, 53706, USA

[3] Cellular and Molecular Biology Training Program, University of Wisconsin, Madison, WI, 53706, USA

[4] Department of Ophthalmology and Visual Sciences, University of Wisconsin-Madison, Madison, WI 53706

[5] McPherson Eye Research Institute, University of Wisconsin, Madison, WI, 53706, USA

[6] Current address: Wisconsin Institute for Science Education and Community Engagement, University of Wisconsin, Madison, WI, 53706, USA

[7] Current address: Molecular and Cellular Biology Program and Department of Genome Sciences, University of Washington, Seattle, WA 98195-5065

[8] Current address: Promega Corporation, 2800 Woods Hollow Road, Madison, WI 53711

* Correspondence to ygrinblat@wisc.edu

Supplementary Figure 1. Single antibody controls for rsph9/ ace-tubulin immunostaining (controls for bleed-through)

Wild type embryos were stained with an acetylated α-tubulin (A) or anti-Rsph9 antibody (B). Embryos were then incubated with DAPI (cyan) to label nuclei.

A-A'': pronephric duct in embryo stained for α-tubulin only. Note absence of ciliary staining on the 568 channel when overexposed.

B-B": Pronephric duct in embryo stained for Rsph9 only. Note absence of ciliary staining on the 488 channel, when overexposed.

Supplementary Figure 2. Western blot analysis of Rsph9 antibody reactivity.

A: Protein alignment shows strong conservation between zebrafish, human and mouse Rsph9 orthologs (predicted MW 30.9 kD). Asterisk marks position of frame shift caused by mutations in rsph9^{uw208} and rsph9^{uw212}. B, C: Western blots of whole-embryo lysates from 4-5-dpf embryos, stained with anti-Rsph9 antibody. B: lysates from a group of rsph9$^{208/208}$ and rsph9^{208}/+ siblings (lane 1) and wild type (lane 2) siblings contain several bands that cross-react with the antibody. Only one band migrates at the predicted ~31 kD size of rsph9 protein. Note reduced intensity of the band in heterozygous/homozygous embryos. C: lysates from rsph9$^{208/208}$ (lane 1) and rsph9$^{208/+}$ (lane 2) siblings. The ~ 31 kD band is absent in rsph9^{208}/ rsph9^{208} embryos, while the higher bands are independent of the rsph9 genotype. Asterisk marks the predicted position of zebrafish Rsph9 protein. MW=PrecisionPlus Protein staindards, BioRad.

Supplementary Figure 3. Rsph9 genotyping assay.

DNA was extracted from immunostained 1dpf embryos after confocal imaging (A), or 4dpf larvae (B). Rsph9 CRISPR site was PCR-amplified and analyzed on a 3.5% Metaphor gel. A: rsph9^{uw208} genotyping results. B: rsph9^{uw212} genotyping results. PCR products were digested with MboII prior to electrophoresis.

Supplementary Figure 4. Morpholino designed against the translation start site of Rsph9 blocks translation from exogenous Rsph9 RNA.

A-D: Wild-type embryos injected with different combinations of rsph9 MO and rsph9-tRFP mRNA. A: Injection of rsph9-tRFP mRNA alone results in tRFP fluorescence at varying levels between embryos. B: Co-injection of rsph9MO with tRFP mRNA does not prevent tRFP fluorescence. C,D: Co-injection of rsph9MO with rsph9-tRFP mRNA, which contains the binding site of the rsph9MO, prevents expression of rsph9-tRFP, as indicated by absence of RFP fluorescence.

Supplementary Figure 5. Morpholino knockdown of Rsph9 causes structural axonemal defects.

A-H: TEM images of ciliary cross-sections in rsph9 morphant embryos at 1 dpf. Ventral neural cilia with 9+0 axonemes found in the ventral midbrain (A) and spinal cord (B) of uninjected control embryos. Note outer dynein arms (arrow), indicative of ciliary motility. Rsph9-depleted morphants contain aberrant 8+1 cilia in the ventral midbrain (C, D). Pronephric cilia in uninjected controls have 9+2 axonemes (E). Rsph9 morphants contain a mixture of cilia with 9+2 (F), supernumerary central microtubules, e.g. 9+5 (G) and 9+0 (H). I: Summary of TEM analysis of ciliary axonemes in wildtype, control morpholino-injected, and Rsph9 morpholino-injected embryos.

Supplementary Figure 6. Dorsal cilia are uniform across brain subdivisions and throughout neurulation. Fixed *Tg(β-actin:mGFP)* embryos stained with antibodies against acetylated α-tubulin (green), gamma-tubulin (blue) and GFP (red). (A,B) Cilia in the dorsal FB do not change in length over time. (C) Average length of dorsal FB cilia at 12S (1.36μm), and 24S (1.02μm). (D,E) Dorsal MB cilia remain unchanged over time. (F) Averaged length at 12S (1.53μm) and 24S (1.54μm). (G,H) Dorsal HB cilia remain

unchanged over time. (I) Averaged length at 12S (0.98μm) and 24S (1.07μm). Cilia length was measured in Image J (NIH). Averages were compiled from 20 cilia picked from 10um stacks of the dorsal- and ventral-most regions of the neural tube.
A-B, D-E, G-H are stacked confocal images with anterior to the left. Error bars represent standard error of the mean.

Supplementary Figure 7. Distinct subpopulations of ventral cilia in each brain subdivision increase in length during neurulation. *Tg(β-actin:mGFP)* embryos stained with antibodies against acetylated α-tubulin (green), gamma-tubulin (blue) and GFP (red), except in A, where nuclei are stained red. (A-C) Cilia in the ventral FB do not change in length over time. (D) Average length of ventral FB cilia at 12S (1.36μm), 18S (1.87μm) and 24S (1.69μm). (E-G) Ventral MB cilia are longer than primary cilia on average and increase in length over time. (H) Average length at 12S (2.76μm), 18S (4μm) and 24S (5.77μm). (I-K) Ventral HB cilia are longer than MB cilia at 12S and increase in length over time. (L) Average length at 12S (3.72μm), 18S (5.02μm) and 24S (5.01μm). A-C, E-G, I-K are stacked confocal images with anterior to the left. Error bars represent standard error of the mean.

Supplementary Figure 8. Rsph9 immunoreactivity co-localizes with acetylated tubulin in hair cell kinocilia.

Wildtype embryos at 3 dpf were immunostained for acetylated tubulin (yellow) and rsph9 (magenta). A-A'': crista hair cells with kinocilia labeled by acetylated tubulin and rsph9. B-B'': macula hair cells with kinocilia labeled by acetylated tubulin and rsph9.

Supplementary Figure 9. Rsph9 function is not required for kinocilia formation or hair cell mechanotransduction.

rsph9^{208}/+ (A,C) and in rsph9^{208}/rsph9^{208} (B,D) sibling embryos at 3 dpf were immunostained for acetylated tubulin to label kinocilia. A, B: kinocilia of the inner ear lateral crista hair cells. C, D: kinocilia in the lateral line neuromasts.

E, F: larvae were derived from a cross between rsph9^{208}/ rsph9^{208} females and rsph9^{208}/+ males and treated with FM1-43 vital dye at 6 dpf. E (higher magnification in E'): a representative FM1-43 treated rsph9^{208}/+ larva. F (higher magnification in F'): a representative FM1-43 treated rsph9^{208}/rsph9^{208} larva.

Supplementary Figure 1:

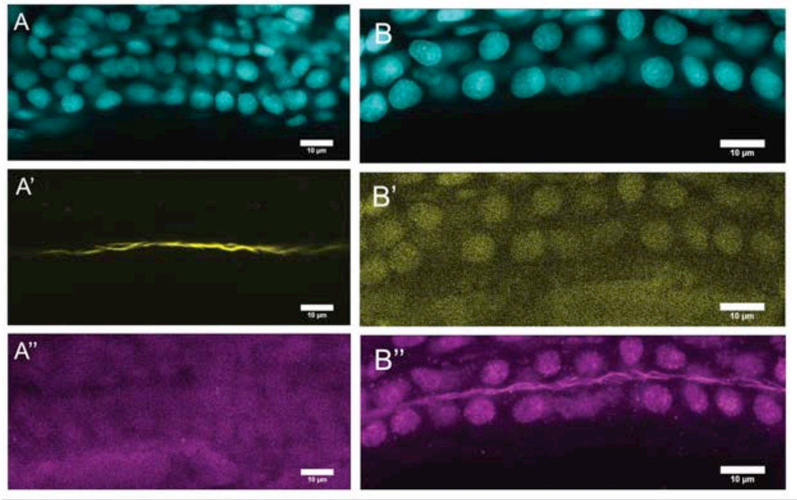

Supplementary Figure 2:

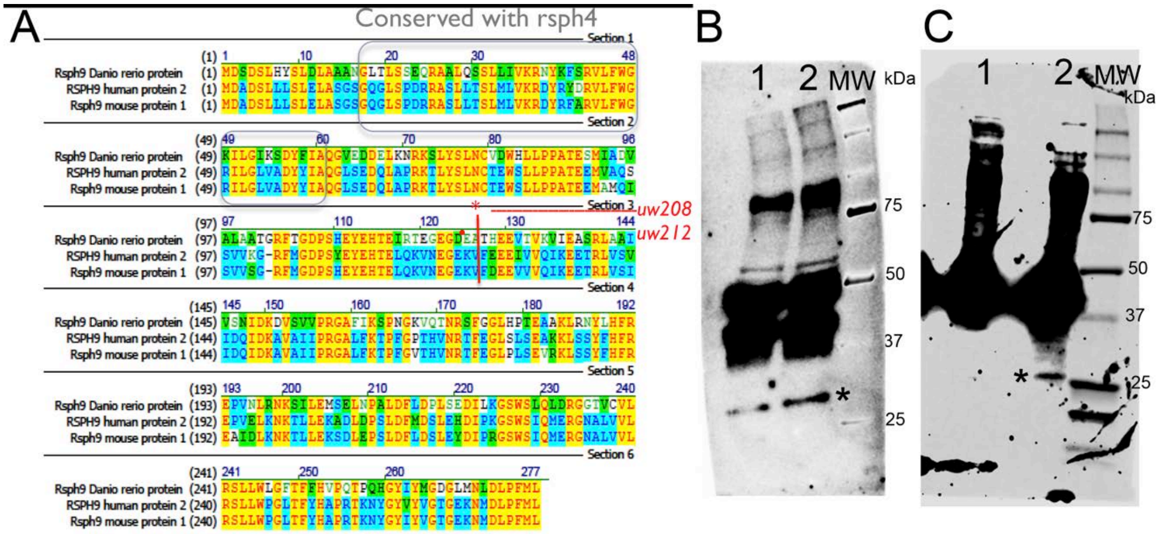

Supplementary Figure 3:

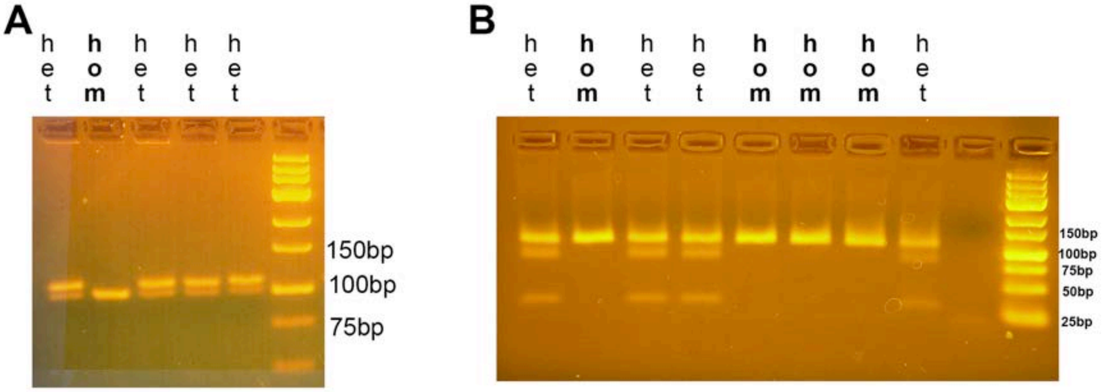

Supplementary Figure 4:

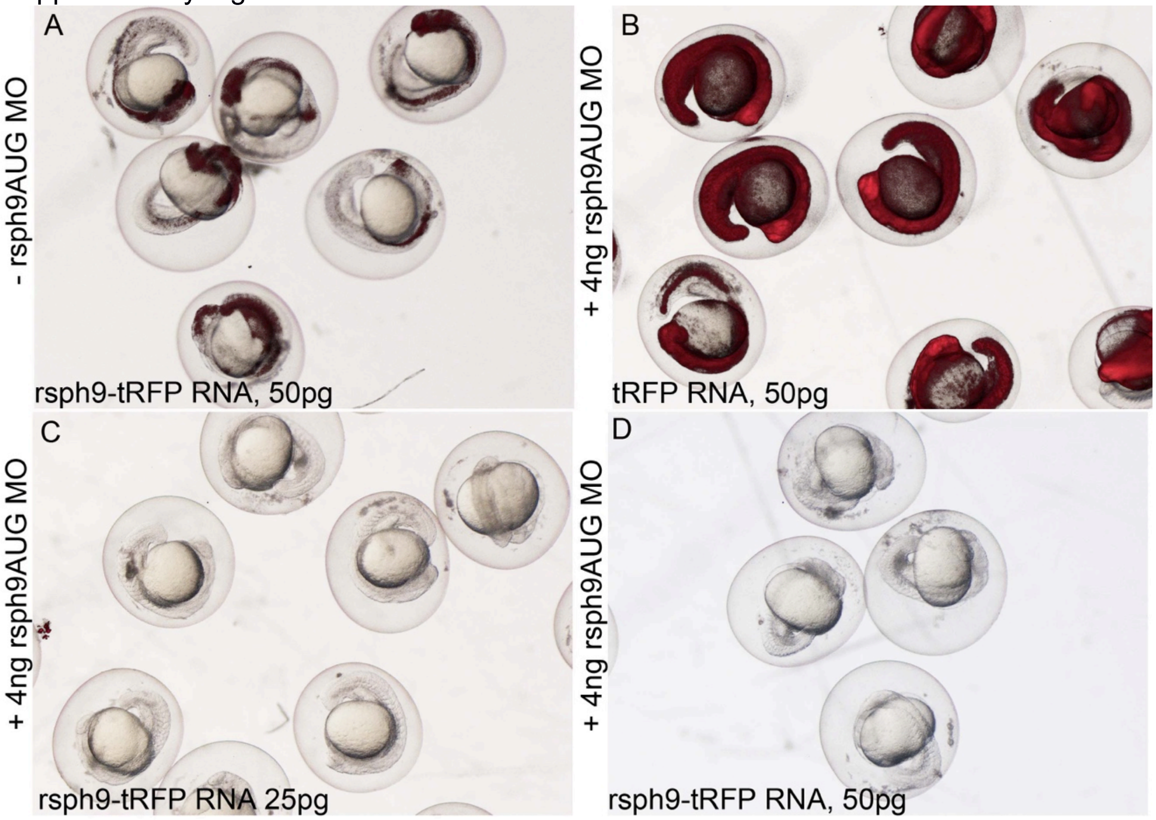

Supplementary Figure 5:

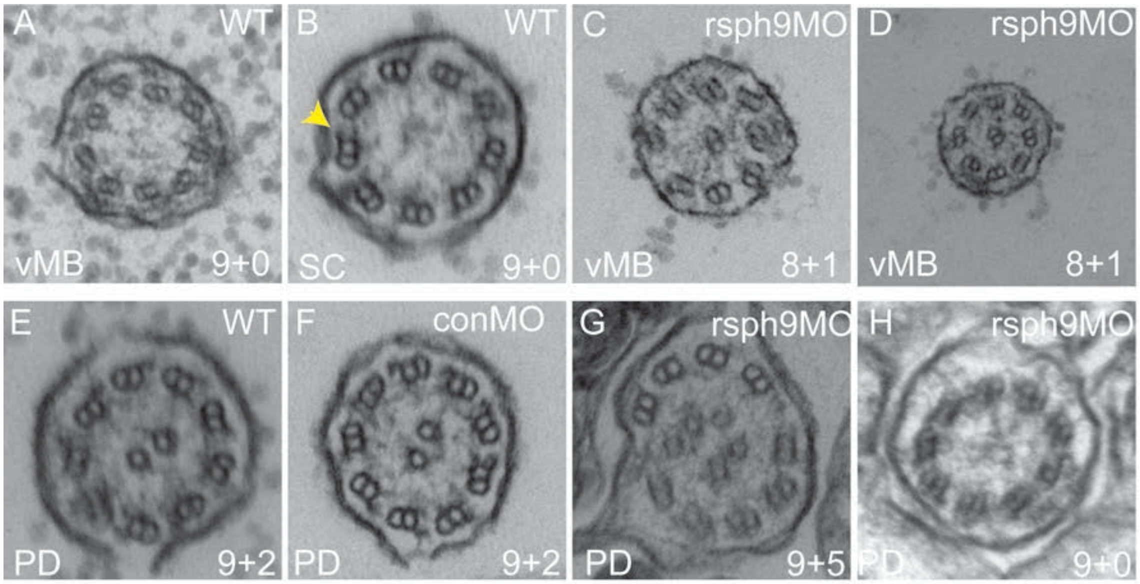

I

		9+2	9+0	8+1	9+(1,4,5)	Total
ConMO	Ventral MB	0	11	1	0	12
Rsph9MO	"	0	6	3	0	9
ConMO	Kidney	16	0	0	0	16
Rsph9MO	"	10	5	0	7	22

Supplementary Figure 6:

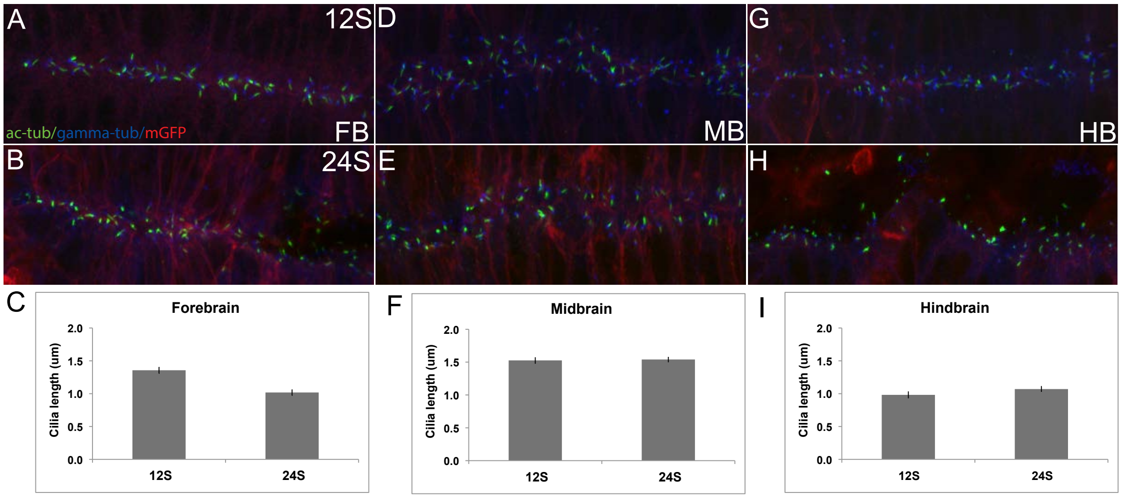

Supplementary Figure 7:

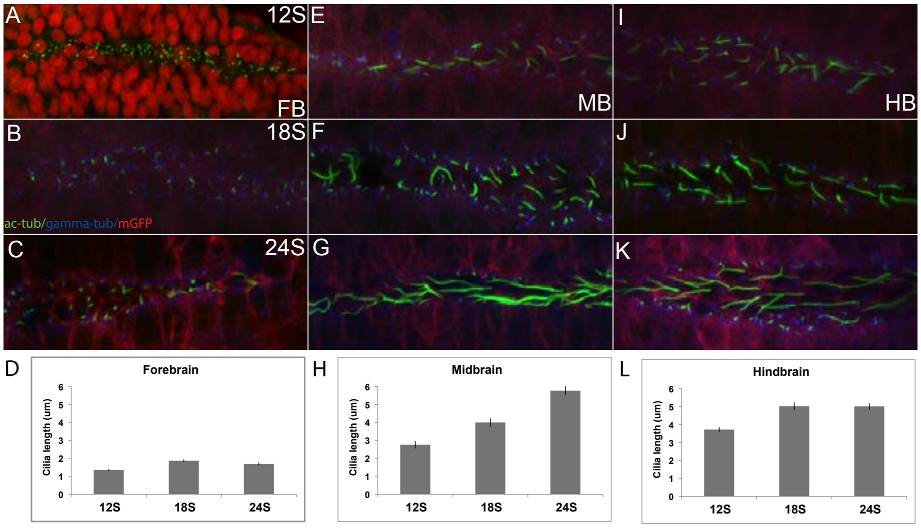

Supplementary Figure 8:

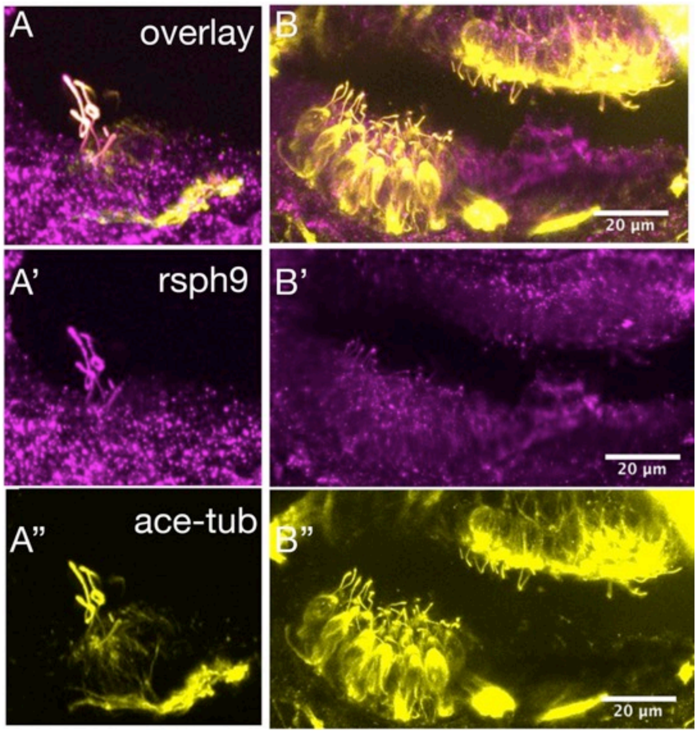

Supplementary Figure 9:

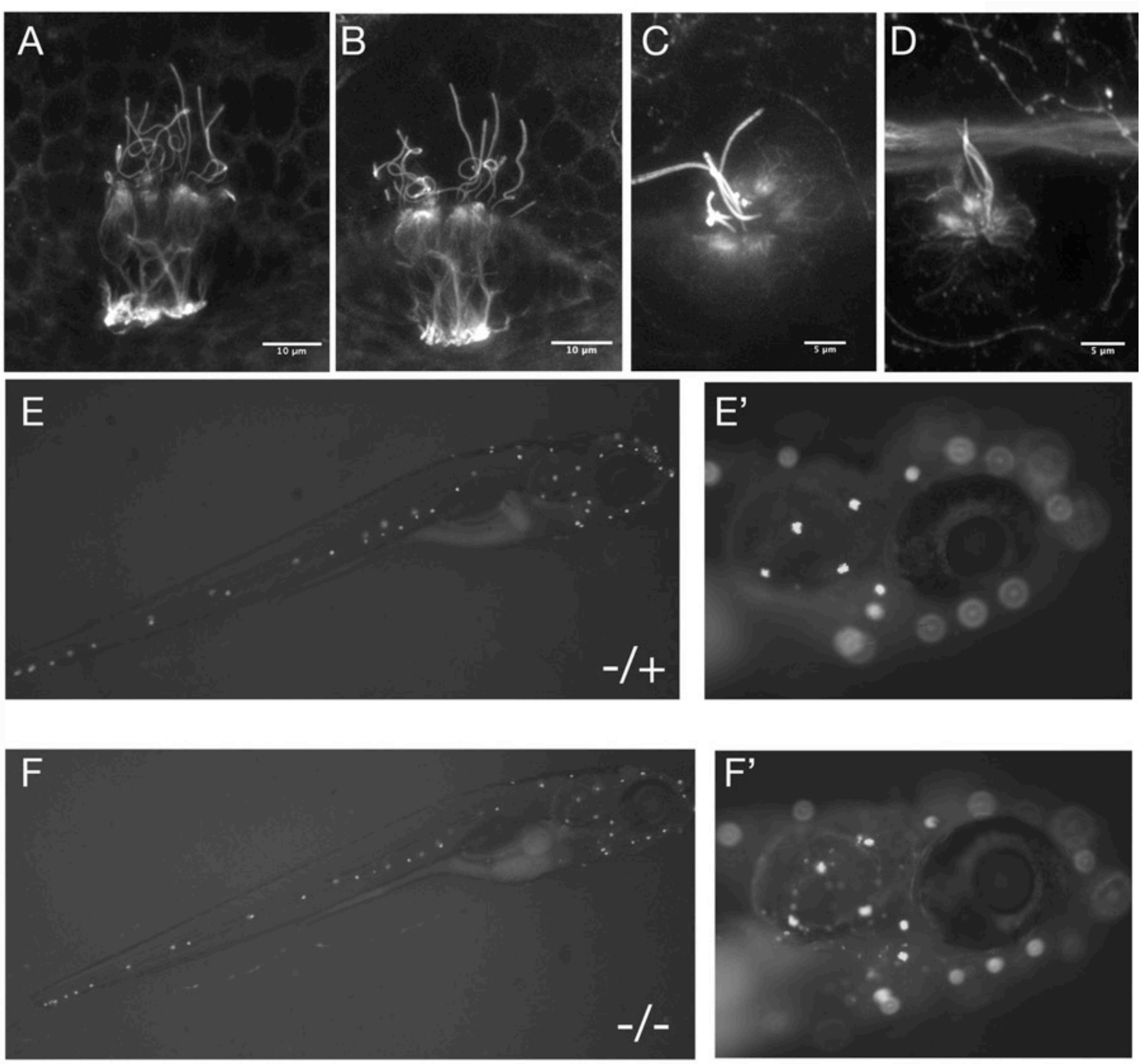

Table 1. Summary of scoring results and larval genotypes for *in vivo* imaging of olfactory cilia.

8-7-15 4dpf F1s from rsph9 ^{208} hom females x het males			
**Embryo #**	**Movie File**	**Genotype**	**Vigorous coordinated motility**
1	IMG_0105	het	+
2	IMG_0106	het	+
3	IMG_0107	het	+
4	IMG_0108	homoz	-
5	IMG_0109	homoz	-
6	IMG_0110	het	+
7	IMG_0111	het	+
8	IMG_0113	homoz	-
9	IMG_0114	het	+

8-10-15 4dpf F1s from rsph9 ^{208} het females x hom males			
**Embryo #**	**Movie File**	**Genotype**	**Vigorous coordinated motility**
1	IMG_0143	het	+
2	IMG_0144	homoz	-
3	IMG_0145	homoz	-
4	IMG_0146	homoz	-
5	IMG_0147	homoz	+
6	IMG_0148	het	+
7	IMG_0149	het	+
8	IMG_0150	homoz	-
9	IMG_0151	het	+
10	IMG_0152	het	+

Table 2. Summary of scoring results for *in vivo* imaging of spinal cord cilia

Scorer A

	Cilia groups				
**Embryo#**	**motile, regular**	**motile, irregular**	**vibrational**	**Total # of scored cilia**	**Genotype**
**1**	1	1	6	**8**	het
**2**	11	10	3	**24**	het
**8**	4	6	12	**22**	het
**3**	0	0	8	**8**	homoz
**7**	0	0	7	**7**	homoz

Scorer B

	Cilia groups				
**Embryo#**	**motile, regular**	**motile, irregular**	**vibrational**	**Total # of scored cilia**	**Genotype**
**1**	1	1	7	**9**	het
**2**	12	10	2	**24**	het
**8**	4	5	13	**22**	het
**3**	0	0	12	**12**	homoz
**7**	0	0	7	**7**	homoz

Table 3. Summary of acoustic startle response initiation analysis.

ASR EXPERIMENT		LOW STIMULUS		HIGH STIMULUS			
RSPH9^{208}	**MEAN**	**LOW-SLC**	**LOW-LLC**	**HIGH-SLC**	**HIGH-LLC**	**N**	
	WT	23.03214286	58.10357143	77.15	88.58148148	28	
	HET	19.23714286	64.9	76.37714286	83.428125	35	
	MUT	7.663636364	20.17272727	31.95454545	51.08181818	22	$p<0.001$ for all MUT means vs W
	**SEM**	**LOW-SLC**	**LOW-LLC**	**HIGH-SLC**	**HIGH-LLC**		
	wt	1.583796061	2.034610186	1.517995772	3.747989928		
	het	1.318833729	1.762277939	1.718682009	3.5358847		
	mut	1.395749934	1.57106258	3.037653409	3.382706812		
RSPH9^{212}	**MEAN**	**LOW-SLC**	**LOW-LLC**	**HIGH-SLC**	**HIGH-LLC**	**N**	
	wt	21.58846154	58.31153846	73.23461538	92.5	26	
	het	25.07727273	60.41590909	79.61363636	88.935	44	
	mut	6.842105263	27.9	25.85789474	49.15789474	19	$p<0.001$ for all MUT means vs W
	**SEM**	**LOW-SLC**	**LOW-LLC**	**HIGH-SLC**	**HIGH-LLC**		
	wt	2.045618357	2.076121977	3.087968613	2.186917624		
	het	0.874774351	1.695848802	1.7862354	2.460523693		
	mut	1.718138832	2.061587526	2.638779578	3.273630266		

Supplementary Movies.

OC1-4: live imaging of olfactory cilia. See Supplementary Table 1 for details.

SC1- 5: live imaging of spinal cord cilia. Representative movies from heterozygous embryos #3 (SC1), #7 (SC2), and heterozygous siblings #2 (SC3), #1 (SC4) and #8 (SC5). See Supplementary Table 2 for details.
